# Supplementary material for: Downregulation of Exosomal hsa-miR-551b-3p in Obesity and Its Link to Type 2 Diabetes Mellitus
Source: Noncoding RNA. 2023 Nov 2;9(6):67. doi: 10.3390/ncrna9060067 (PMC10660712; doi:10.3390/ncrna9060067)
Supplement: Supplementary file 1 [file ncrna-09-00067-s001.zip › Table S1.pdf]

Table S1. Primers for miRNA detection using reverse transcription and real time PCR.

microRNA	Specific RT-primer and PCR reverse primer
hsa-miR-551b-3p	RT_primer 5’-TGGCATTGATCTGGTTCATCAAGGCCTTGCGACCCATAAAACCAAG-3’<br>REV: 5’-AAACCAAGTATGGGTCGC-3’
hsa-miR-302d-3p	RT_primer 5’-TGGCATTGATCTGGTTCATCAAGGCCTTACTTTAACATGTGCCTCC-3’<br>REV: 5’-CATGTGCCTCCATGTTAAAGT-3’
hsa-miR-145-5p	RT_primer 5’-TGGCATTGATCTGGTTCATCAAGGCCTTGTCCAGTTTTTCCTGGG-3’<br>REV: 5’-TTCCTGGGAAAACTGGAC-3’
hsa-miR-132-3p	RT_primer 5’-TGGCATTGATCTGGTTCATCAAGGCCTTACCGTGGCTTACAATCGA-3’<br>REV: 5’-AACAATCGAAAGCCACGGT-3’
hsa-miR-10a-5p	RT_primer 5’-TGGCATTGATCTGGTTCATCAAGGCCTTTACCCTGTAGATTCGGAT-3’<br>REV: 5’-GATTCGGATCTACAGGGTA-3’
hsa-miR-1246	RT_primer 5’-TGGCATTGATCTGGTTCATCAAGGCCTTAATGGATTTTCTGCTCCA-3’<br>REV: 5’-TTCTGCTCCAAAAATCCATT-3’
hsa-miR-378g-3p	RT_primer 5’-TGGCATTGATCTGGTTCATCAAGGCCTTACUGGGCUUGTCTGACTC-3’<br>REV: 5’-GTCTGACTCCAAGCCCAGT-3’
hsa-let-7c-5p	RT_primer 5’TGGCATTGATCTGGTTCATCAAGGCCTTTGAGGTAGTAGCATACAAC-3’<br>REV: 5’-GCATACAACCTACTACCTCA-3’

Universal forward primer	5’-TGGCATTGATCTGGTTCATC-3’
