# Supplementary figures and images for: Downregulation of Exosomal hsa-miR-551b-3p in Obesity and Its Link to Type 2 Diabetes Mellitus
Source: Noncoding RNA. 2023 Nov 2;9(6):67. doi: 10.3390/ncrna9060067 (PMC10660712; doi:10.3390/ncrna9060067)

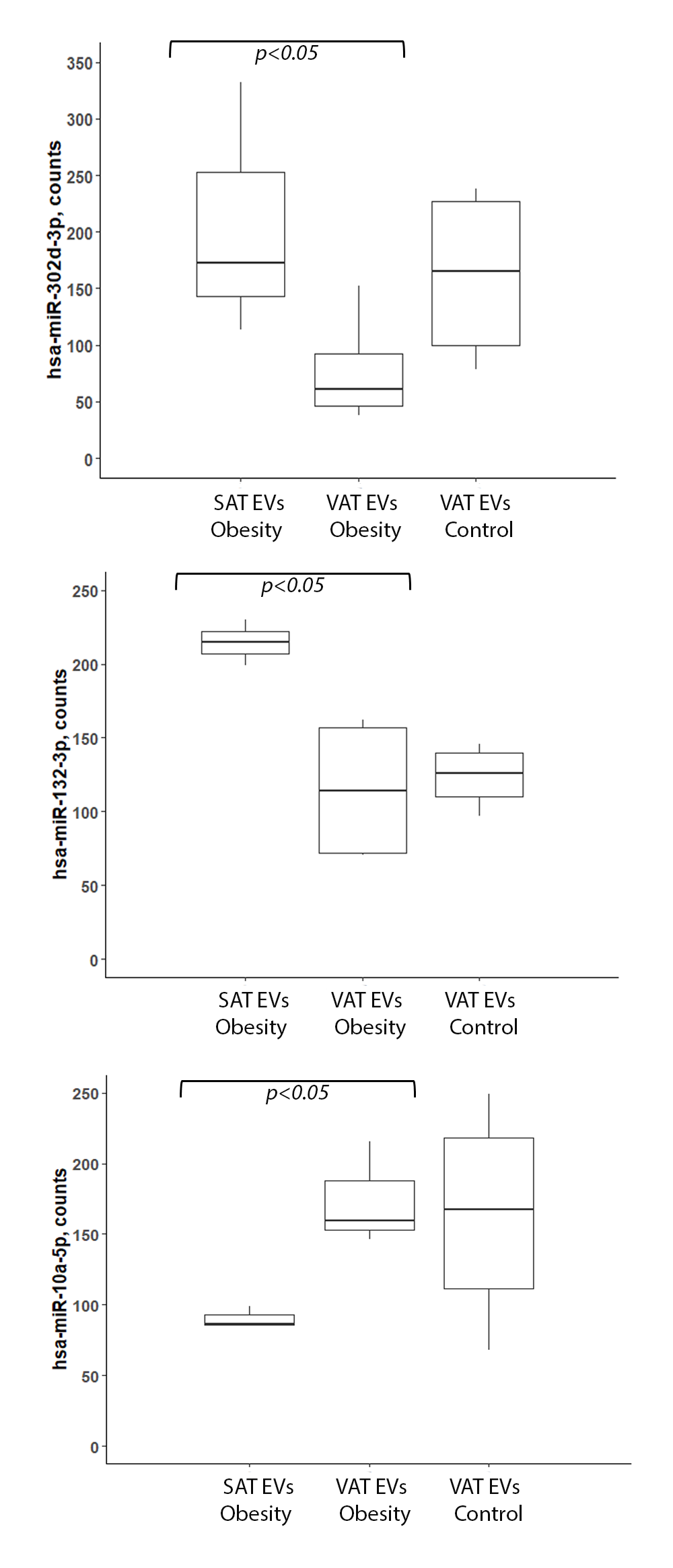

Supplement: Supplementary file 1 [file ncrna-09-00067-s001.zip › figure S1.tif]

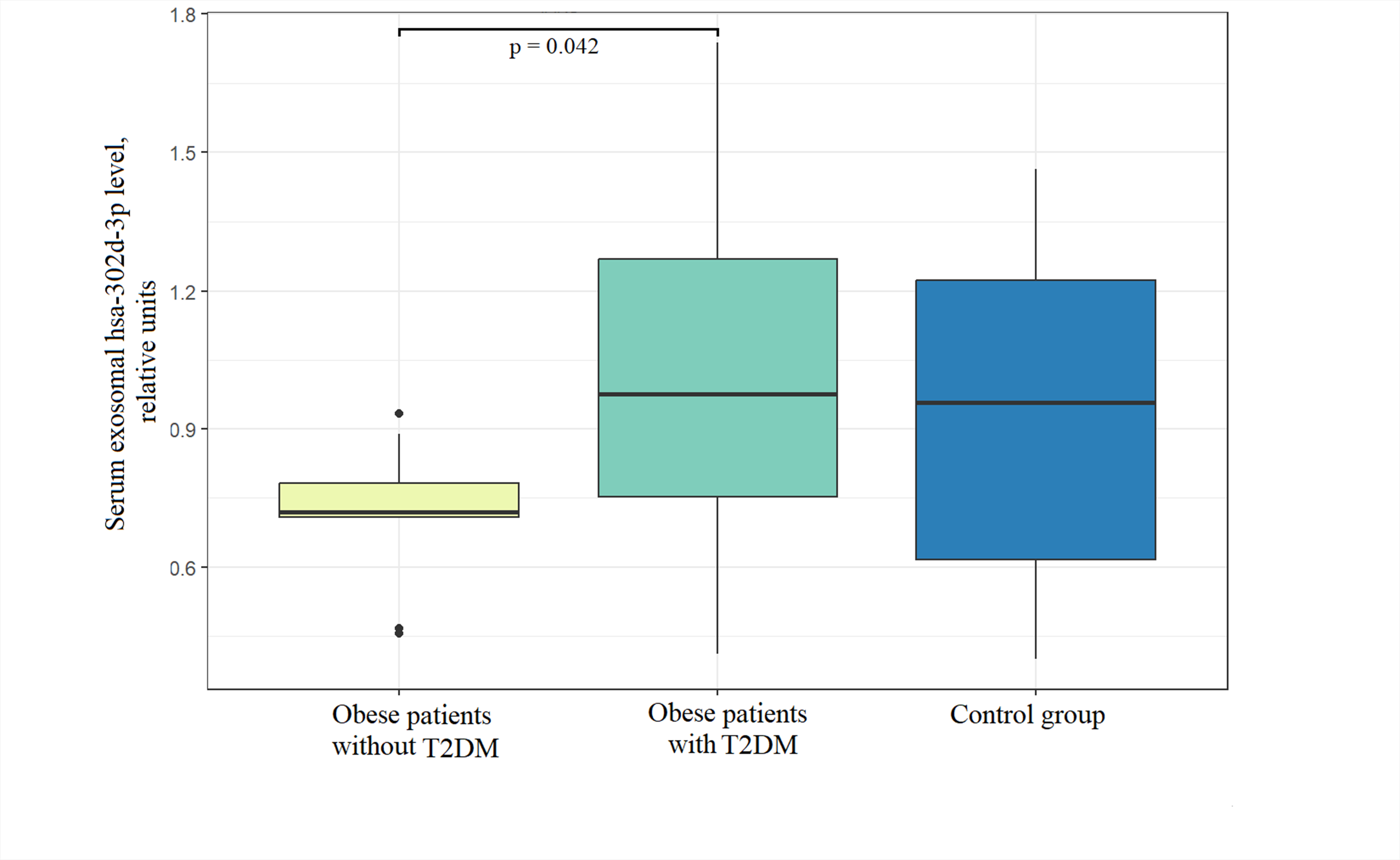

Supplement: Supplementary file 1 [file ncrna-09-00067-s001.zip › figure S2.tif]
